# Enhancing ^19^F Benchtop NMR Spectroscopy by Combining *para*-Hydrogen Hyperpolarization and Multiplet Refocusing

**DOI:** 10.1021/acsmeasuresciau.2c00055

**Published:** 2022-11-08

**Authors:** Ana I. Silva Terra, Matheus Rossetto, Claire L. Dickson, George Peat, Dušan Uhrín, Meghan E. Halse

**Affiliations:** † Department of Chemistry, University of York, YorkYO10 5DD, U.K.; ‡ EaStCHEM School of Chemistry, University of Edinburgh, EdinburghEH9 3FJ, U.K.

**Keywords:** benchtop NMR spectroscopy, SABRE hyperpolarization, NMR sensitivity enhancement, SHARPER, ^19^F NMR

## Abstract

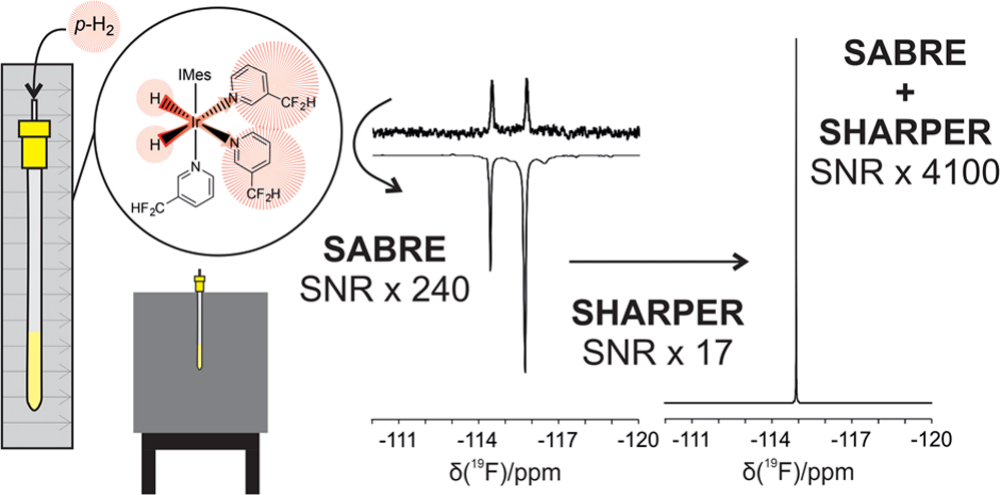

Benchtop NMR spectrometers provide a promising alternative to high-field NMR for applications that are limited by instrument size and/or cost. ^19^F benchtop NMR is attractive due to the larger chemical shift range of ^19^F relative to ^1^H and the lack of background signal in most applications. However, practical applications of benchtop ^19^F NMR are limited by its low sensitivity due to the relatively weak field strengths of benchtop NMR spectrometers. Here we present a sensitivity-enhancement strategy that combines SABRE (Signal Amplification By Reversible Exchange) hyperpolarization with the multiplet refocusing method SHARPER (Sensitive, Homogeneous, And Resolved PEaks in Real time). When applied to a range of fluoropyridines, SABRE-SHARPER achieves overall signal enhancements of up to 5700-fold through the combined effects of hyperpolarization and line-narrowing. This approach can be generalized to the analysis of mixtures through the use of a selective variant of the SHARPER sequence, *sel*SHARPER. The ability of SABRE-*sel*SHARPER to simultaneously boost sensitivity and discriminate between two components of a mixture is demonstrated, where selectivity is achieved through a combination of selective excitation and the choice of polarization transfer field during the SABRE step.

## Introduction

In recent years, benchtop NMR spectrometers with moderate fields of 1–2 T and line widths of <0.5 Hz, have emerged as a promising alternative to traditional high-field NMR spectrometers for applications where instrument size and/or cost are a limiting factor and for use in nontraditional environments, such as within a fume cupboard in a synthetic lab.^1−5^ While benchtop NMR spectrometers benefit from increased portability and lower purchase and maintenance costs, they suffer from reduced sensitivity and chemical shift dispersion due to their lower magnetic field strengths.^6^ The resolution challenge of benchtop NMR is particularly acute for protons because of their narrow chemical shift range, which leads to signal overlap and strong coupling effects.

Fluorine NMR is an attractive alternative as it has a wider chemical shift range than ^1^H and is the most sensitive of all spin-1/2 nuclei other than ^1^H, due to its 100% natural abundance and high gyromagnetic ratio. Furthermore, ^19^F NMR is of particular analytical interest due, for example, to the high prevalence of fluorine in pharmaceuticals,^7−9^ the use of fluorinated tags in chemical biology,^10^ and reaction monitoring applications.^11^^19^F is a convenient target in benchtop NMR because fluorine measurements can usually be performed using the proton channel due to the proximity of the ^19^F and ^1^H Larmor frequencies at 1–2 T. Despite the sensitivity challenges, benchtop ^19^F NMR has been investigated for practical applications such as monitoring the degradation of persistent fluorinated organic pollutants.^12^

One strategy to overcome the sensitivity challenge of benchtop NMR is to use hyperpolarization, which increases the population difference between nuclear spin states and, as a result, the detected signal. *para*-Hydrogen-based hyperpolarization methods, such as the Signal Amplification By Reversible Exchange (SABRE)^13^ method, are particularly attractive for use with compact NMR devices because they do not require large or expensive instrumentation and the level of polarization generated is independent of the detection field strength.^14^ This has led to the combination of SABRE with benchtop NMR detection by many groups to deliver enhancements of several orders of magnitude for a range of nuclei, including ^1^H, ^13^C, ^15^N, and ^19^F.^15−27^

**Figure 1:**
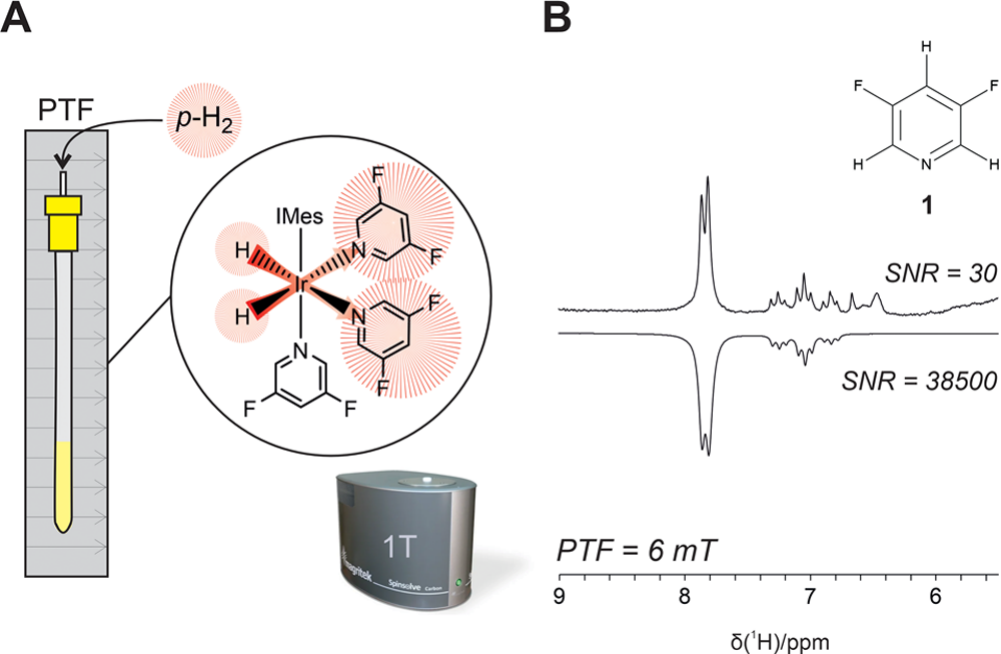
(A) Diagram showing the SABRE hyperpolarization process followed by measurement using benchtop NMR. *para*-Hydrogen is added to the headspace of an NMR tube containing the SABRE catalyst and target substrate. It is then shaken in the desired PTF to allow for a buildup of hyperpolarized substrate in free solution. Finally, the sample is introduced into a benchtop NMR spectrometer for detection. B) Comparison of a nonhyperpolarised (top) and a SABRE-enhanced (bottom) ^1^H NMR spectrum of 3,5-difluoropyridine (**1**) zoomed in between 6.5 and 9 ppm. SABRE hyperpolarization (PTF = 6 mT) provides a 1200-fold improvement in the single-scan signal-to-noise ratio (SNR).

In SABRE, the high spin order of the nuclear singlet isomer of hydrogen, *para*-hydrogen (*p*-H_2_), is harnessed to create highly polarized states in a target molecule through reversible binding to an iridium catalyst (Figure 1A).^13^ Reversible binding of both *p*-H_2_ and the target analyte (the substrate) to the complex establishes a temporary *J* coupling network through which spontaneous transfer of the polarization can occur. This transfer is facilitated by a weak magnetic field, referred to as the polarization transfer field (PTF). The optimal PTF varies according to the specific spin system and is typically in the range of 0 < PTF ≤ 10 mT.^28,29^ In the presence of the relevant PTF and under a continual supply of *p*-H_2_, substrate hyperpolarization is built up in free solution over a period of seconds due to the chemical exchange of both the substrate and *p*-H_2_.^30^ Enhanced NMR spectra are observed following introduction of the sample into the NMR spectrometer for detection (Figure 1B). The chemical identity of the substrate is not altered in this process and so it can be rehyperpolarised by adding fresh *p*-H_2_.^13^

Another approach to improving the sensitivity of ^19^F NMR is the multiplet refocusing method SHARPER (Sensitive, Homogeneous, And Resolved PEaks in Real time).^31^ SHARPER increases signal-to-noise ratios (SNR) by eliminating peak-splitting through the use of FID acquisition periods interleaved with a series of 180° refocusing pulses. Importantly, this is achieved by applying radio frequency (RF) pulses only at the frequency of the acquired nucleus. An additional benefit of the SHARPER approach is that it minimizes the effect of field inhomogeneity, producing narrow peaks that approach their natural line width. We recently introduced adapted versions of SHARPER and the chemically selective variant, *sel*SHARPER, for use on benchtop NMR spectrometers that remove the need for pulsed field gradients (PFG), which are not available on many benchtop instruments.^32^ When combined with optimized data processing strategies, including a matched filter, ^19^F SNR improvements by more than an order of magnitude were achieved.

The goal of this work is to optimize the sensitivity of ^19^F benchtop NMR spectroscopy through the combination of SABRE and SHARPER. To explore the efficacy of this approach, we first optimize the ^19^F SABRE enhancement of three fluoropyridines using benchtop NMR detection and a range of PTFs. The additional SNR gains that can be achieved by combining SHARPER with SABRE are demonstrated and compared to a standard ^1^H decoupling approach. Finally, a modified version of the *sel*SHARPER experiment is applied to a mixture of two analytes to establish the ability of SABRE-SHARPER to deliver both SNR enhancement and resonance discrimination.

## Experimental Section

**Scheme 1:**
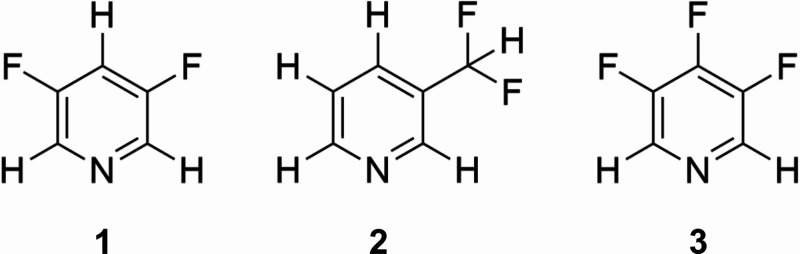
3,5-Difluoropyridine (**1**), 3-(Difluoromethyl)pyridine (**2**), and 3,4,5-Trifluoropyridine (**3**)

Single component SABRE samples contained 100 mM of the substrate (**1**, **2**, or **3** in Scheme 1) and 5 mM of precatalyst [IrCl(COD)(IMes)] (where COD: 1,5-cyclooctadiene and IMes: 1,3-bis(2,4,6-trimethyl-phenyl)-imidazolium) in 0.7 mL of HPLC grade methanol. The exact masses used in the preparation are provided in the SI (Table S1). All samples were sonicated to aid dissolution and then introduced into a 5 mm NMR tube with a J-Young valve and degassed with 3 cycles of freeze/pump/thaw using a Schlenk line and an acetone and dry ice bath. The degassing step is included to improve the dissolution of *p*-H_2_ and to remove dissolved oxygen. The headspace of the NMR tubes were then filled with 4 bar of *p*-H_2_, which was generated by cooling H_2_ gas to 28 K to produce >99% *p*-H_2_ using a generator described previously.^14^ For each SABRE acquisition, the sample was shaken for 10 s inside the desired polarization transfer field (PTF) and then was introduced into the spectrometer for data acquisition. Typical sample transfer times were on the order of 2 s. The desired PTF was achieved by shaking the sample (a) inside a hand-held magnetic array with an average field PTF = 6.2 mT,^33^ (b) in the ambient Earth’s magnetic field adjacent to the spectrometer (PTF ∼ 50 μT), or (c) inside a μ-metal shield that reduces the ambient field by a factor of ∼300-fold to achieve a PTF ∼ 0.2 μT. Between each SABRE measurement, the *p*-H_2_ in the sample was refreshed by evacuating the headspace of the NMR tube and refilling with fresh *p*-H_2_. For each fresh sample, the filling-shaking-acquiring process was repeated four to six times to activate the SABRE catalyst. The **1** and **2** SABRE mixture samples were prepared using the same procedure with 50 mM of each compound, respectively (Table S1). Each SABRE measurement was repeated three times. The SABRE SNR values were calculated as the average of the three repetitions and are reported with the corresponding standard error.

Benchtop NMR spectra were acquired using a 43 MHz (1 T) NMR spectrometer (Spinsolve Carbon, Magritek, Aachen, Germany) equipped with ^1^H/^19^F and ^13^C channels. Shimming and frequency calibrations were performed on a reference sample containing a H_2_O:D_2_O mixture (10%:90%) before data was collected. Data was acquired using the Spinsolve Expert software (version 1.41) and processed either by Prospa (version 3.63), MestReNova (version 14.1.2.25024) or Matlab (version R2020a).

Unless otherwise stated, all of the SHARPER and *sel*SHARPER spectra were acquired in a single scan using an acquisition chunk time of τ = 3.2 ms within each echo loop and a 180° pulse duration of 225 μs. Complete sets of pulse sequence parameters for all experiments are provided in the SI (Section S2).

Free induction decay data were apodized with a matched exponential decay filter, zero-filled and Fourier transformed to produce the spectra, which were manually phased and baseline corrected as needed. For non-SHARPER acquisitions, a filter was chosen that optimized SNR without significantly compromising spectral resolution. For full details see the SI (Section S2). For all SHARPER and *sel*SHARPER spectra, the imaginary signal channel was zeroed prior to Fourier Transformation to improve SNR, as described previously.^32^

Signal-to-noise values were calculated as the ratio of the signal height of the tallest peak and the standard deviation of a region of spectral noise. When reference spectra were obtained by averaging more than one scan, the inverse of the square root of the number of scans was used as the correction factor to obtain a signal-to-noise ratio per one scan. The SNR enhancement factors (ε_*SNR*_) were calculated as the ratio of the SNR of the hyperpolarised spectrum and the SNR per one scan of the reference spectrum.

## Results and discussion

A range of fluoropyridines, including 3,5-difluoropyridine (**1**), have been shown previously to provide efficient ^1^H and ^19^F SABRE hyperpolarization when combined with high-field NMR detection.^17,34,35^ Therefore, the three fluorinated pyridines selected for this work, 3,5-difluoropyridine (**1**), 3-(difluoromethyl)pyridine (**2**), and 3,4,5-trifluoropyridine (**3**) (Scheme 1), are expected to deliver good levels of SABRE enhancement. In addition, the chosen substrates provide three different ^19^F coupling environments to test the efficacy of the SABRE-SHARPER approach. Specifically, **1** has a single ^19^F resonance with a medium strength heteronuclear coupling constant of ^3^*J*_HF_ = 9 Hz, **2** has a single ^19^F resonance with a larger heteronulcear coupling constant of ^2^*J*_HF_ = 55 Hz, and **3** contains two ^19^F resonances with both homonuclear and heteronuclear *J* couplings.

**Figure 2:**
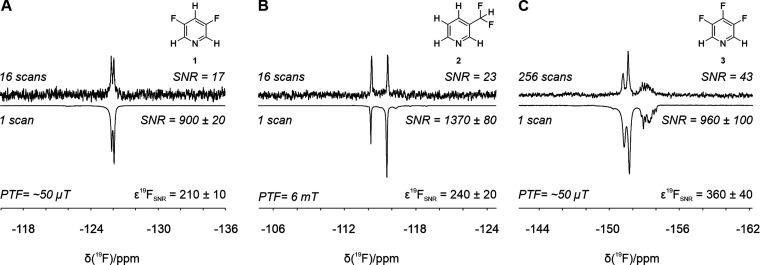
Comparison of SABRE-enhanced and thermally polarized ^19^F benchtop NMR spectra of 100 mM of (A) **1**, (B) **2**, and (C) **3** and 5 mM precatalyst in methanol. All SABRE spectra were acquired with a single scan. The vertical scale of the thermal spectra was increased to aid visualization. SABRE SNR values are reported as the average over three repeat measurements. Fluorine SNR enhancement factors (ε_SNR_) are calculated as the ratio of the average SNR of the SABRE spectra and the SNR per one scan of the reference spectrum.

SABRE-enhanced spectra of **1**, **2**, and **3** were recorded using PTF = 6.2 mT and benchtop (1 T) NMR detection (Figure S1). ^1^H SNR enhancements of 1200-fold, 600-fold, and 1400-fold were observed for **1**, **2**, and **3**, respectively, indicating efficient SABRE activity for all three species. To optimize polarization transfer to ^19^F, three different PTF regimes were tested for each compound: PTF = 6.2 mT, PTF = 50 μT (the ambient Earth’s magnetic field outside the spectrometer) and PTF ∼ 0.2 μT (field within a μ-metal shield). Optimized SABRE-enhanced ^19^F benchtop NMR spectra for **1**, **2**, and **3** are presented in Figure 2, with thermally polarized NMR spectra included for reference.

Increases in ^19^F SNR by factors of 210 ± 10, 240 ± 20, and 360 ± 40 were observed for **1**, **2**, and **3**, respectively (Figure 2). Interestingly, while the optimal PTF for **1** and **3** was the Earth’s magnetic field (EF ∼ 50 μT), PTF = 6.2 mT was found to be most efficient for **2**. The level anticrossing (LAC) theory of efficient SABRE polarization transfer suggests that the optimal PTF for ^19^F lies in the μT regime, while direct transfer to ^1^H is optimized in mT fields.^28,29,36^ This framework is consistent with the findings of Chukanov et al.,^34^ who studied the SABRE polarization transfer mechanisms for 3-fluoropyridine and observed more efficient transfer to ^19^F in microtesla fields, such as the Earth’s magnetic field, compared to transfer in mT fields. In the case of **2** we observe much higher enhancements in the mT regime than in the Earth’s field. This suggests that ^19^F is polarized indirectly, with polarization being transferred first to ^1^H nuclei on the substrate and then spin-relayed to ^19^F, with the transfer within the substrate being mediated by the large heteronuclear coupling constant, ^2^*J*_*HF*_ = 55 Hz.

**Figure 3:**
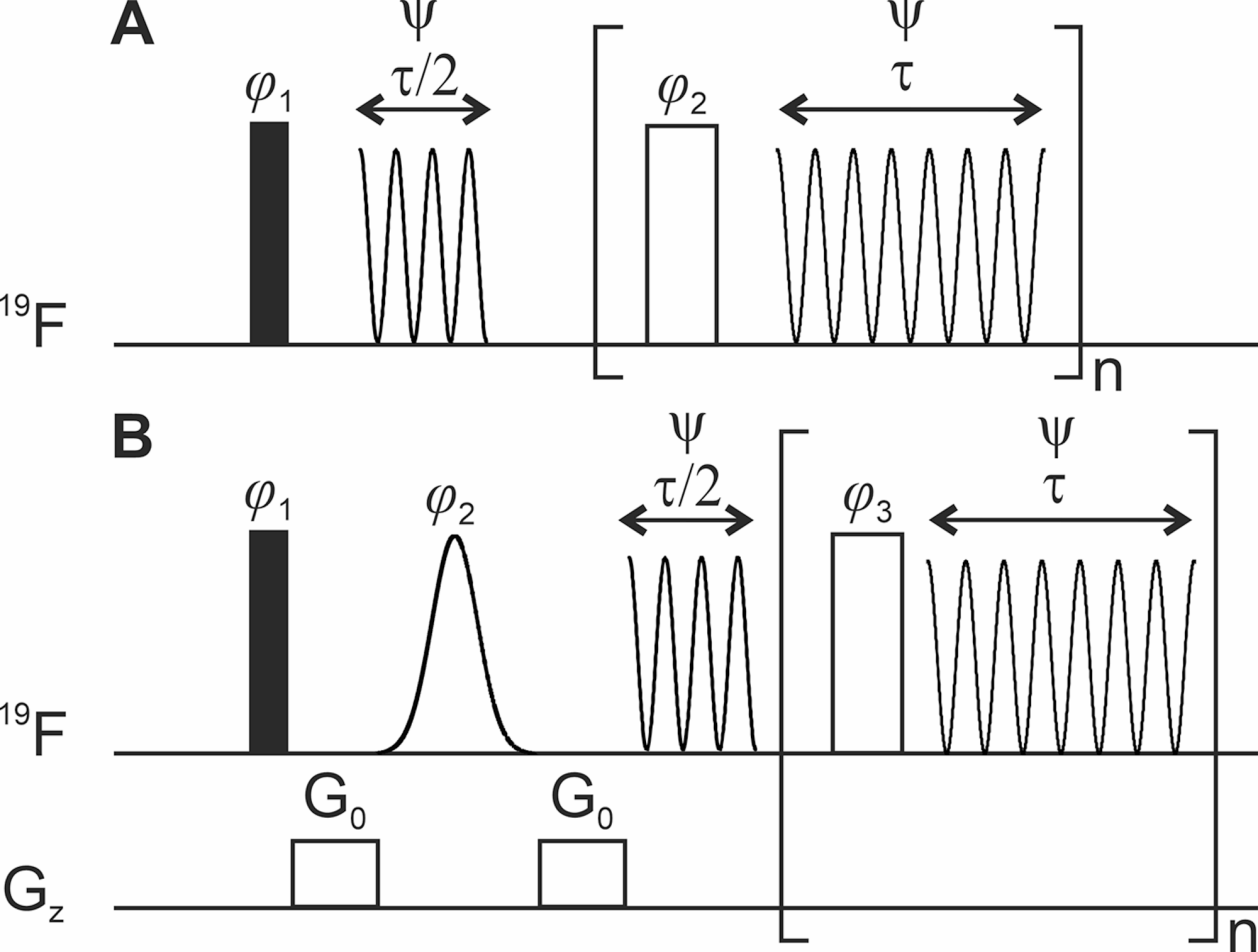
Pulse sequence diagrams for (A) SHARPER and (B) *sel*SHARPER with nonselective pulses in the pulse train. The filled and empty rectangles represent 90° and 180° hard pulses, while the smoothed empty shape depicts a selective Gaussian 180° pulse. The chunk time is defined as τ = *Nδt*, where *N* is the number of points per chunk and *δt* is the dwell time. The total acquisition time is *t*_acq_ = (*n* + 1/2)τ, where *n* is the total number of loops, (A) SHARPER: φ_1_ = *x*; φ_2_ = *y*; ψ = *x*. (B) *sel*SHARPER φ_1_ = *x*; φ_2_ = y; φ_3_ = −*y*; ψ = *x*.

Inspection of the SABRE-enhanced ^19^F NMR spectra in Figure 2 suggests that significant further ^19^F SNR enhancements are possible by removing inhomogeneous peak broadening and collapsing the multiplets into a single resonance using the SHARPER approach. Figure 3a presents the SHARPER pulse sequence developed for use on benchtop NMR spectrometers that does not rely on the use of pulsed field gradients within the sampling loop.^32^ In the first step of the experiment, a broadband 90° pulse is applied and a first half chunk of the free induction decay (FID) is acquired. This is followed by a CPMG-style loop that includes additional short acquisition chunks interleaved with 180° refocusing pulses. The complete FID is assembled by combining the acquisition chunks acquired within each spin–echo period. In this way, for on-resonance spins, all heteronuclear *J* couplings and inhomogeneous broadening are refocused, leading to a single narrow peak.

A consequence of the piece-wise collection of the FID is the appearance of sideband artifacts at multiples of the inverse of the chunk time, τ = *Nδt*, where *N* is the number of points per chunk and *δt* is the dwell time. These artifacts are minimized for short chunk times, which do not allow for significant chemical shift or *J* coupling evolution. However, very short sampling intervals limit the overall SNR gains that can be achieved because the time spent applying the refocusing pulses increases as a proportion of the total acquisition time. Therefore a balance is required to achieve clean spectra with high SNR.^31,32^

Usually, optimal performance with minimal artifacts is achieved in multiple scans through the use of phase cycling. However, single scan methods are preferable for combining with SABRE because of the transient nature of the hyperpolarization. While we exclusively use single scans for the SABRE-SHARPER experiments presented here, automated methods for moving the sample between the polarization transfer and detection fields, via flow or sample shuttling for example, have been shown to enable multistep experiments, including SABRE-enhanced 2D benchtop NMR spectroscopy.^15,16,37,38^ These multidimensional experiments could be combined with the SABRE-SHARPER approach presented here to provide a wider range of structural and dynamic information.

**Figure 4:**
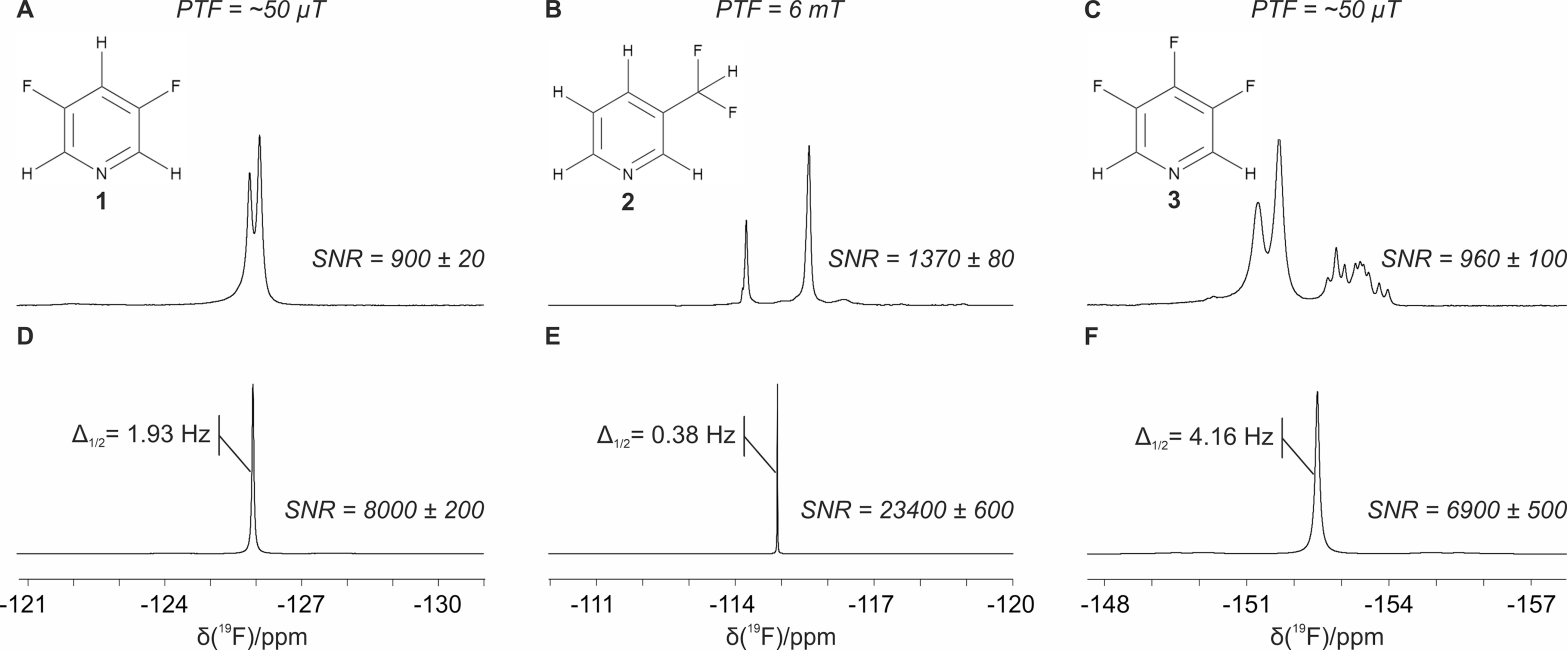
Comparison of (A–C) SABRE and (D–F) SABRE-SHARPER ^19^F benchtop (1 T) NMR spectra of 100 mM **1**, **2** or **3** and 5 mM SABRE precatalyst in methanol. All spectra were acquired in a single scan and were apodized by a matched exponential filter (see Table S3). The SNR values represent averages over three repeat experiments. Full width at half-maximum values are shown for the SABRE SHARPER spectra. We note that these line-widths are effectively double the fundamental SHARPER line width due to the application of the matched filter. Individual traces were magnified as required to aid visualization.

Figure 4 presents a comparison of ^19^F SABRE spectra (A–C) with corresponding SABRE-SHARPER spectra (D–F) for **1**, **2**, and **3**, respectively. A relatively short acquisition period of τ = 3.2 ms was used in each loop of the SHARPER sequence. In all cases, the SHARPER sequence has succeeded in collapsing the spectra into a single resonance. While all SABRE-SHARPER spectra demonstrate SNR improvements, the extent of enhancement varies for each molecule: 8.9-fold for **1**, 17-fold for **2**, and 7.2-fold for **3**. The differences in SHARPER efficacy are also evident in the line widths achieved in each case. We note that due to the use of a matched exponential filter to optimize SNR, the observed line width is approximately double the fundamental line-width achieved by the SHARPER sequence. The effective  relaxation times and corresponding line-widths  for all SHARPER experiments are provided in the Supporting Information (Section S3).

In the case of **1**, the SABRE-SHARPER sequence achieves a line width of  = 0.97 Hz prior to the application of the matched filter. When SHARPER is applied to the sample without SABRE hyperpolarization, a much narrower line width of  = 0.18 Hz is achieved (Figure S2A). This indicates that the nature of the hyperpolarization generated by SABRE is interfering with the optimal performance of SHARPER in this case. It is well established that, since the origin of the hyperpolarization in SABRE is the singlet state of *p*-H_2_, higher-order spin states can be enhanced alongside single-spin magnetization.^39^ Therefore a possible explanation for the imperfect refocusing of the SHARPER sequence is interference from higher-order terms (e.g., two-spin order ^19^F–^1^H states) that behave differently under the train of refocusing pulses. Nevertheless, even with imperfect refocusing, an overall SNR advantage of 1900-fold is obtained by the combined effects of SABRE and SHARPER.

In the case of **2**, SABRE-SHARPER achieves an excellent line width of  = 0.19 Hz prior to apodization, which is comparable to the performance of SHARPER without SABRE hyperpolarization (Figure S2B). Thus, for **2**, the combination of SABRE and SHARPER results in an overall SNR advantage relative to the thermally polarized spectrum of 4100-fold.

**Figure 5:**
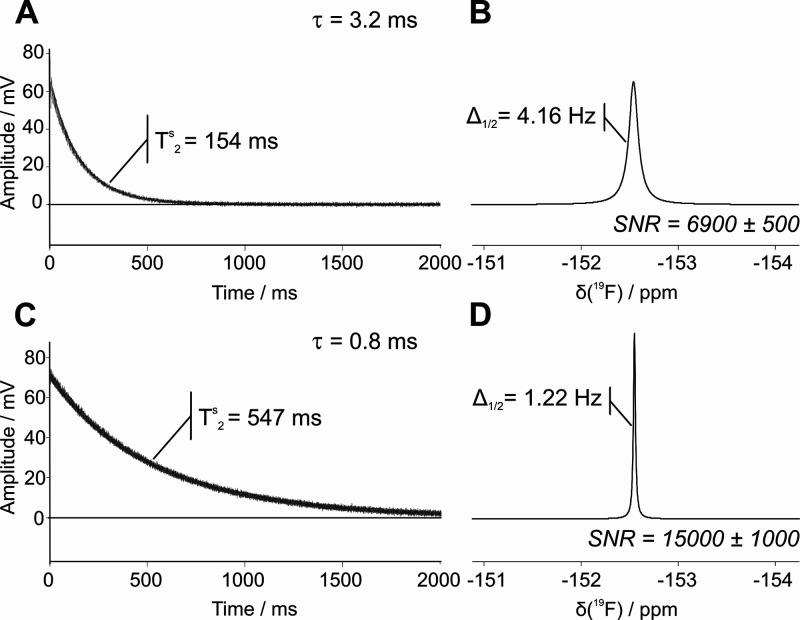
Comparison of ^19^F SABRE-SHARPER FIDs (A,C) and spectra (B,D) of 100 mM of **3** with 5 mM SABRE precatalyst in methanol, acquired with chunk lengths of τ = 3.2 ms (A,B) and τ = 0.8 ms (C,D). Spectra were acquired in a single scan and were apodized by matched exponential filters (see Table S3). The SNR values represent averages over three repeat experiments. Full width at half-maximum values are shown for the SABRE-SHARPER spectra and include the effects of apodization.

In the case of **3**, the SHARPER sequence efficiently refocuses the chemical shift difference between the two ^19^F resonances (1.9 ppm = 75 Hz) as well as the heteronuclear (*J*_HF_) and to some extent the homonuclear (*J*_FF_) couplings. As with **1**, the line width achieved prior to apodization for **3**,  = 2.1 Hz, is much broader than the expected natural line width. However, here it is comparable to what is achieved for this sample using SHARPER without SABRE hyperpolarization (Figure S2C). Unlike **1** and **2**, the spin system in **3** contains a significant homonuclear coupling of ^3^*J*_FF_ = 18 Hz, which will continue to evolve during the τ = 3.2 ms period. In order to improve the efficiency of the line-narrowing, we repeated the SHARPER and SABRE-SHARPER experiments on **3** with a much shorter chunk time of τ = 0.8 ms. We note that the use of very short delays between RF pulses is more straightforward to achieve on a benchtop NMR spectrometer, when compared to high-field NMR, because the lower Larmor frequency requires lower RF power and therefore less stringent duty cycle constraints on long trains of RF pulses. Figure 5 presents a comparison of the SABRE-SHARPER FIDs acquired with τ = 3.2 ms and τ = 0.8 ms. We observe a significant increase in the apparent relaxation time from  = 154 ms to  = 547 ms. This corresponds to a decrease in line width from  = 2.1 Hz to  = 0.58 Hz, prior to apodization, and a corresponding increase in the SNR advantage of SABRE-SHARPER relative to SABRE from 7.2-fold to 16-fold. Overall, SABRE-SHARPER with τ = 0.8 ms provides a total SNR increase of 5700-fold relative to a standard ^19^F acquisition. We note that in the case of **1** and **2**, no additional SNR improvements were observed when shorter chunk lengths were used (τ < 3.2 ms).

An alternative strategy to boost SNR and simplify the ^19^F peaks is ^1^H decoupling. However, this is not straightforward to implement on the benchtop NMR instrument because ^19^F and ^1^H share the same coil and so heteronuclear ^1^H decoupling can only be implemented in an interleaved manner. In practice we find that, while SABRE-enhanced ^19^F {^1^H} benchtop NMR spectra are simplified, no net SNR gain is observed (see Figure S3). In addition, the decoupling conditions were challenging to optimize and led to significant peak and baseline distortions in many cases.

A limitation of the SHARPER approach for the analysis of mixtures is that it collapses all signals into a single peak and therefore does not distinguish between components. This can be addressed by using the selective variant, *sel*SHARPER,^31^ in which the broadband excitation is replaced with a single pulsed-field gradient spin–echo (SPFGSE) element. On the Magritek Spinsolve benchtop NMR spectrometer used in this work, we are able to use the first-order shims to provide the pulsed-field gradient (PFG) pulses required for selective excitation. However, if no PFG is available, we have proposed alternative selection strategies previously.^32^

In the original version of *sel*SHARPER, the rectangular pulses inside the acquisition loop are replaced by selective shaped pulses in order to maximize selectivity and refocus homonuclear couplings.^31^ However, we found that for the SABRE hyperpolarised samples explored here, this was not required to achieve efficient refocusing due to the short echo times that can be achieved on the benchtop spectrometer. Furthermore, the introduction of shaped pulses within the loop significantly reduced the observed SNR due to the time required to apply the refocusing pulses as a proportion of the total acquisition time. Therefore, we have implemented a variant of *sel*SHARPER, shown in Figure 3B, where nonselective pulses are used in the loop. By applying this simplified variation of *sel*SHARPER, it is possible to isolate the signal of interest in a mixture and boost its SNR in a single scan. If multiple components are of interest, the sequence can be repeated once for each target resonance. An additional benefit of this approach, is that selectivity can be improved without incurring an SNR penalty by increasing the duration of the initial selective RF pulse without changing the nonselective RF pulses inside the refocusing loop.

**Figure 6:**
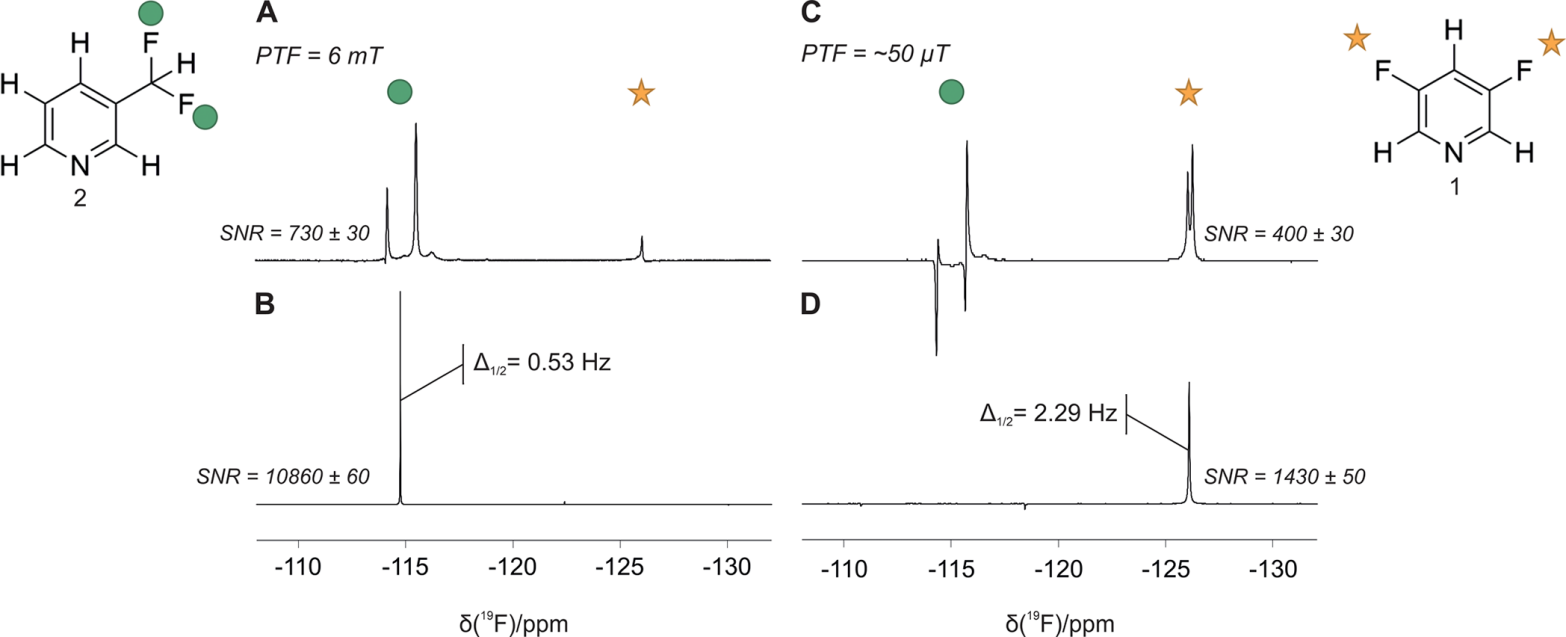
Comparison between SABRE-enhanced ^19^F benchtop NMR spectra with (A,C) standard and (B,D) *sel*SHARPER acquisition for a mixture containing **1** and **2**. SABRE spectra were acquired with (A,B) PTF = 6.2 mT and (C,D) PTF ∼ 50 μT. SABRE-*sel*SHARPER spectra were acquired with selection of **2** in B and selection of **1** in D. All spectra were acquired in a single scan and were apodized by a matched exponential filter (see Table S3). The SNR values represent averages over three repeat experiments. Full width at half-maximum values are shown for the SABRE-*sel*SHARPER spectra. Vertical scales were increased as required to aid visualization.

To evaluate the performance of SABRE combined with *sel*SHARPER, a sample containing 50 mM each of **1** and **2** was analyzed. As these compounds have different optimal PTF values, we are able to achieve an element of selectivity from both SABRE and SHARPER. In addition, the resonances for these compounds are separated by 11.1 ppm (∼ 450 Hz at 1 T) and therefore provide a good test for the selectivity of the *sel*SHARPER sequence. The results of combining SABRE and *sel*SHARPER are shown in Figure 6, where SABRE hyperpolarization in Figure 6A and B was achieved using PTF = 6.2 mT, which is optimal for **2**, while PTF ∼ 50 μT was used for Figure 6C and D to optimize the signal of **1**.

Considering first the effect of the PTF, we observe that for PTF = 6.2 mT (Figure 6A), the enhancement of **2** is maximized while **1** is significantly reduced relative to Figure 6C. In the case of PTF ∼ 50 μT, the SABRE enhancement of **1** is optimized; however, the enhancement of **2** is not suppressed. Instead, we observe strongly antiphase signals for 2, which are characteristic of SABRE enhancement of a two-spin-order term, rather than the desired in-phase single-spin magnetization.^39^ Nevertheless, in both cases, as observed in Figure 6B and D, the combination of SABRE with *sel*SHARPER successfully isolates and narrows the target signal from the mixture, while increasing the SNR by factors of 3.6-fold and 15-fold, for **1** and **2**, respectively.

When the original *sel*SHARPER sequence,^32^ with 5 ms selective pulses inside the loop, is applied to the mixture sample under SABRE conditions, there was a significant sensitivity penalty that led to SNR enhancements of only 2.7-fold and 7.3-fold for **1** and **2**, respectively (Figure S4). This reduction in SNR performance relative to the results in Figure 6 is due to the different way the couplings are removed in the original selective SHARPER experiments, where longer selective pulses within the acquisition loop accelerate the apparent *T*_2_ relaxation, causing signal broadening and hence lower SNR. Therefore, *sel*SHARPER with nonselective pulses in the loop and a short chunk time, τ (Figure 3b), is the preferred method for optimizing SNR with the combined SABRE-*sel*SHARPER approach.

Another popular strategy for removing peak splittings is to use pure shift techniques to simplify spectra via broadband homonuclear decoupling.^40^ A benefit of pure shift approaches is that chemical shift information is retained; however, this generally comes at the cost of signal loses, which, depending on the method, can be as much as 75–90%.^40^ Furthermore, the widely used PSYCHE (pure shift yielded by chirp excitation) methods,^41^ which retain ca. 25% of the ^1^H NMR signal, use a pseudo 2D acquisition strategy that requires multiple acquisitions to produce a 1D spectrum and therefore cannot easily be combined with single-shot SABRE experiments. An exception is the HOBS (homodecoupled band selective) method,^42^ which provides additional sensitivity and can, in principle, be acquired in a single scan. A limitation of this approach, particularly for benchtop NMR applications where chemical shift dispersion is limited by the lower magnetic field strength, is the requirement for chemical shift separation between the coupled peaks. Additionally, the standard pure shift methods do not compensate for magnetic field inhomogeneity. Two pure shift methods that are designed to generate high resolution spectra in the presence of field inhomogeneity have been introduced.^43,44^ However, these are pseudo 2D methods that cannot be acquired in a single scan. Therefore, if the goal is to maximize the SNR in a single shot SABRE experiment, SHARPER is the superior approach, as it achieves homo- and heteronuclear decoupling at no expense to sensitivity in a single scan while compensating for magnetic field inhomogeneity. Additionally, multiresonance SHARPER acquisition can be achieved and has been applied to reaction monitoring followed by simultaneous acquisition of one signal from each of the reactant and product.^45^

All of the SABRE-SHARPER spectra presented here were acquired in a single scan immediately following SABRE hyperpolarization. However, if there are significant background nonhyperpolarized signals present, these can give rise to off-resonance artifacts in the SHARPER spectra. Such signals can be efficiently removed by acquiring a reference nonhyperpolarised second scan (Figure S5). However, this second scan will necessarily decrease the overall SNR as it adds in extra noise but no further hyperpolarised signal. Therefore, it should only be used if the artifacts introduced by any background signals overlap or interfere with the target signal in the SABRE-SHARPER spectrum.

## Conclusions

We have presented an optimized approach to ^19^F benchtop NMR spectroscopy that exploits the combined SNR enhancements of SABRE hyperpolarization and the multiplet-refocusing method, SHARPER. The SABRE-SHARPER method was tested on three fluorinated pyridines, 3,5-difluoropyridine, 3-(difluoromethyl)pyridine, and 3,4,5-trifluoropyridine, which are characterized by a range of homo- and heteronuclear *J* coupling constants and chemical shift differences. Significant ^19^F SNR enhancements of up to 360-fold were obtained by SABRE hyperpolarization in a single scan, following optimization of the polarization transfer field (PTF). The combination of SABRE with SHARPER achieved a further boost in SNR of up to 17-fold by removing inhomogeneous broadening and refocusing homo- and/or heteronuclear *J* couplings and chemical shift differences on the order of 75 Hz. Taken together, the SABRE-SHARPER approach achieved SNR enhancements of up to 5700-fold relative to a standard ^19^F NMR acquisition. SNR increases were not observed when using ^1^H decoupling to remove heteronuclear couplings due to the limitations of the shared ^1^H/^19^F channel. A selective variant of SHARPER was combined with SABRE to enhance the sensitivity of the two components in a mixture of 3,5-difluoropyridine and 3-(difluoromethyl)pyridine, which are separated by only 450 Hz at 1 T. While a separate experiment is required to enhance each component, excellent selectivity and overall SNR enhancements of 630-fold and 4300-fold were achieved for 3,5-difluoropyridine and 3-difluoromethylpyridine, respectively. For analytical applications, the quantification of hyperpolarised NMR signals is a challenge because the observed signal intensity depends on the hyperpolarization efficiency, which is analyte specific and can vary with experimental parameters, including analyte concentration. A route to overcome this has been proposed by Tessari and co-workers.^46^ They have demonstrated a linear dependence of the observed ^1^H SABRE hyperpolarization as a function of analyte concentration for a range of N-heterocycles, where one or more cosubstrates are used to stabilize the active SABRE catalyst.^46,47^ A feature of this method is the requirement that the target analytes are present in substoichiometric concentrations relative to the catalyst. In this way, the limit of detection for SABRE-enhanced analytes can be reduced to submillimolar concentrations. Indeed, Eshuis et al. have reported the detection of SABRE-enhanced ^1^H NMR signals for analyte concentrations on the order of 10 μM using NMR detection at 11.7 T (600 MHz). Combining this strategy with the SABRE-SHARPER approach provides a promising route to achieving submillimolar limits of detection for benchtop ^19^F NMR spectroscopy. In addition, we note that the SHARPER acquisition itself is quantitative, as has been demonstrated previously for its use in reaction monitoring applications.^31^ While we have focused here on ^19^F benchtop NMR, we anticipate that equally significant SNR gains can be achieved by applying the SABRE-SHARPER approach to other low-sensitivity nuclei, such as ^13^C. Equally, we observe comparable SNR gains for ^1^H benchtop NMR spectra with SABRE-SHARPER; however, the ability to selectively enhance a target species in this case is limited by the comparatively narrow chemical shift range for ^1^H.

## Data Availability

The NMR data underlying this study are openly available through the York Research Database at https://doi.org/10.15124/252bc49d-8f1f-4c0d-b1bb-9aeadcdde0c8.

## Supporting Information Available

SABRE sample preparation details; experimental and processing parameters; effective relaxation times, *T*_2_^*S*^, for SHARPER experiments; supplementary figures; implementation of Gaussian pulse with RF nonlinearity correction (PDF)
