# Supplementary material for: Enhancing 19F Benchtop NMR Spectroscopy by Combining para-Hydrogen Hyperpolarization and Multiplet Refocusing
Source: ACS Meas Sci Au. 2022 Nov 8;3(1):73–81. doi: 10.1021/acsmeasuresciau.2c00055 (PMC9936801; doi:10.1021/acsmeasuresciau.2c00055)
Supplement: Supplementary file 1 — tg2c00055_si_001.pdf [file tg2c00055_si_001.pdf]

# Supplementary Data for: Enhancing $^{19}$F benchtop NMR spectroscopy by combining *para*hydrogen hyperpolarisation and multiplet refocusing

Ana I. Silva Terra[1], Matheus Rossetto[1], Claire L. Dickson[2,3], George Peat[2], Dušan Uhrín[2], and Meghan E. Halse*[1]

[1]*Department of Chemistry, University of York, York, YO10 5DD, UK*
[2]*EaStCHEM School of Chemistry, University of Edinburgh, Edinburgh, EH9 3FJ, UK*
[3]*Current address: Oxford Instruments Magnetic Resonance, High Wycombe, HP12 3SE, UK*

**Abstract**

**S1 SABRE sample preparation** **s2**

**S2 Experimental and processing parameters** **s2**

**S3 Effective relaxation times, $T_2^S$, for SHARPER experiments** **s3**

**S4 Supplementary figures** **s4**

**S5 Implementation of Gaussian pulse with RF non-linearity correction** **s5**

*email:meghan.halse@york.ac.uk

# S1 SABRE sample preparation

Single-component SABRE (Signal Amplification By Reversible Exchange) samples of **1** (Sample 1), **2** (Sample 2) and **3** (Sample 3) and SABRE mixture sample of **1** and **2** (Sample 4) were prepared using the masses detailed in Table S1. All samples were prepared in 0.7 mL of protio-methanol.

Table S1: Masses of **1**, **2**, **3** and catalyst used in SABRE sample preparation.

| **Sample** | **Mass of catalyst (mg)** | **Mass of 1 (mg)** | **Mass of 2 (mg)** | **Mass of 3 (mg)** |
|---|---|---|---|---|
| 1 | 2.22 | 7.04 | - | - |
| 2 | 2.33 | - | 8.94 | - |
| 3 | 2.36 | - | - | 8.11 |
| 4 | 2.39 | 3.27 | 4.46 | - |

# S2 Experimental and processing parameters

Full power (0 dB) 90° and 180° pulses required for pulse and collect, $^{19}$F{$^{1}$H} pulse and collect and SHARPER (Sensitive, Homogeneous, And Resolved PEaks in Real time) experiments were 100 $\mu$s and 225 $\mu$s respectively for $^{19}$F measurements and 9.35 $\mu$s and 24.2 $\mu$s for $^{1}$H measurements. We note that the 180° pulse lengths are not twice that for the 90° pulses due to non-linearity of the radio-frequency (RF) amplifier. *Sel*SHARPER experiments used a 5 ms 180° Gaussian pulse generated with 1000 steps and 1% truncation. Details on the calibration of the Gaussian pulse and the scaling used to overcome the non-linearity of the RF amplifier are provided in section S5. Rectangular pulsed field gradients were generated by shifting the shim values along x, y and z to their maximum value for 250 $\mu$s and then reverting to the original values. For $^{19}$F{$^{1}$H} pulse and collect experiments, signal acquisition was interleaved with $^{1}$H 180° inversion pulses such that the total dwell time of 200 $\mu$s for each point consisted of 24.2 $\mu$s for the 180° pulse, a 20 $\mu$s ring-down delay and 155.8 $\mu$s for signal collection.

Typical acquisition parameters for all experiments are summarised in Table S2. The reported acquisition times are for signal acquisition only and do not include delays for RF pulses. All SABRE experiments were carried out in a single scan with no repetition delay. The dwell time for the SHARPER experiments with a chunk time of $\tau = 0.8$ ms was reduced to 50 $\mu$s to accommodate the acquisition of 8 points during the initial half chunk period of $\tau/2 = 0.4$ ms, the minimum allowed by the spectrometer.

Table S2: Acquisition parameters for Pulse and collect,$^{19}$F{$^{1}$H} pulse and collect, SHARPER and *sel*SHARPER experiments.

| Experiment | Number of scans | Repetition time / s | Acquisition time / ms | Dwell time / $\mu$s | $\tau$ / ms |
|---|---|---|---|---|---|
| $^{19}$F Pulse and collect$^{a}$ | 16, 256 | 10 | 6553.6 | 200 | NA |
| $^{1}$H Pulse and collect$^{a}$ | 16, 64 | 10 | 6553.6 | 200 | NA |
| SABRE ($^{19}$F and $^{1}$H) | 1 | - | 6553.6 | 200 | NA |
| SHARPER | 1, 16$^{a}$ | 10$^{a}$ | 6553.6 | 50$^{b}$, 100 | 0.8$^{b}$, 3.2 |
| *sel*SHARPER | 1, 16$^{a}$ | 10$^{a}$ | 6553.6 | 100 | 3.2 |
| SABRE $^{19}$F{$^{1}$H} | 1 | 3 | 819.2 | 200 | NA |

$^{a}$ Thermally-polarised experiments.

$^{b}$ $\tau = 0.8$ ms

For SHARPER experiments, the matched filter exp($-t/T_f$) was chosen to match the experimentally determined effective relaxation times during SHARPER acquisition, $T_2^S$ (see Tables S4 and S5). Filter values for pulse and collect experiments were selected as a compromise between SNR and resolution. For $^{1}$H measurements $T_f = 1000$ ms was used.

Table S3: Filter parameters, $T_f$, applied to $^{19}F$ measurements.

| Experiment | Single-component samples | | | | Mixture sample | |
|---|---|---|---|---|---|---|
| | **1** | **2** | **3** | **3*** | **1** | **2** |
| | $T_f$/ms | $T_f$/ms | $T_f$ /ms | $T_f$ /ms | $T_f$ /ms | $T_f$ /ms |
| Pulse and collect | 500 | 500 | 400 | - | 500 | 500 |
| SHARPER | 1860 | 3330 | 155 | 550 | 1860 | 3330 |
| SABRE | 330 | 1700 | 155 | - | 330 | 1700 |
| SABRE-SHARPER | 330 | 1700 | 155 | 550 | - | - |
| SABRE-*sel*SHARPER[a] | - | - | - | - | 100 | 290 |
| SABRE-*sel*SHARPER | - | - | - | - | 280 | 1160 |

* $\tau = 0.8$ ms

[a] Version with selective pulses inside the loop

## S3 Effective relaxation times, $T_2^S$, for SHARPER experiments

$T_2^S$ values for SHARPER experiments were calculated by fitting the FID (free induction decay) data to a single exponential decay function; the corresponding SHARPER linewidths were calculated as $\Delta_{1/2}^S = 1/\pi T_2^S$.

Table S4: $T_2^S$ and $\Delta_{1/2}^S$ values for single-component samples

| Experiment | **1** | | **2** | | **3** | | **3*** | |
|---|---|---|---|---|---|---|---|---|
| | $T_2^S$ /ms | $\Delta_{1/2}^S$ /Hz | $T_2^S$ /ms | $\Delta_{1/2}^S$ /Hz | $T_2^S$ /ms | $\Delta_{1/2}^S$ /Hz | $T_2^S$ /ms | $\Delta_{1/2}^S$ /Hz |
| SHARPER thermal | 1860±30 | 0.17 | 3330±40 | 0.10 | 146±7 | 2.18 | 470±10 | 0.68 |
| SABRE-SHARPER | 328±2 | 0.97 | 1694 ±2 | 0.19 | 153±2 | 2.07 | 545±2 | 0.58 |

* $\tau = 0.8$ ms

Table S5: $T_2^S$ and $\Delta_{1/2}^S$ values for the mixture sample

| Experiment | **1** | | **2** | |
|---|---|---|---|---|
| | $T_2^S$ /ms | $\Delta_{1/2}^S$ /Hz | $T_2^S$ /ms | $\Delta_{1/2}^S$ /Hz |
| selSHARPER thermal | 3100±100 | 0.10 | 3970±90 | 0.08 |
| SABRE-*sel*SHARPER[a] | 98±1 | 3.24 | 287±1 | 1.11 |
| SABRE-*sel*SHARPER | 277.5±0.4 | 1.15 | 1170±20 | 0.19 |

[a] Version with selective pulses inside the loop.

## S4 Supplementary figures

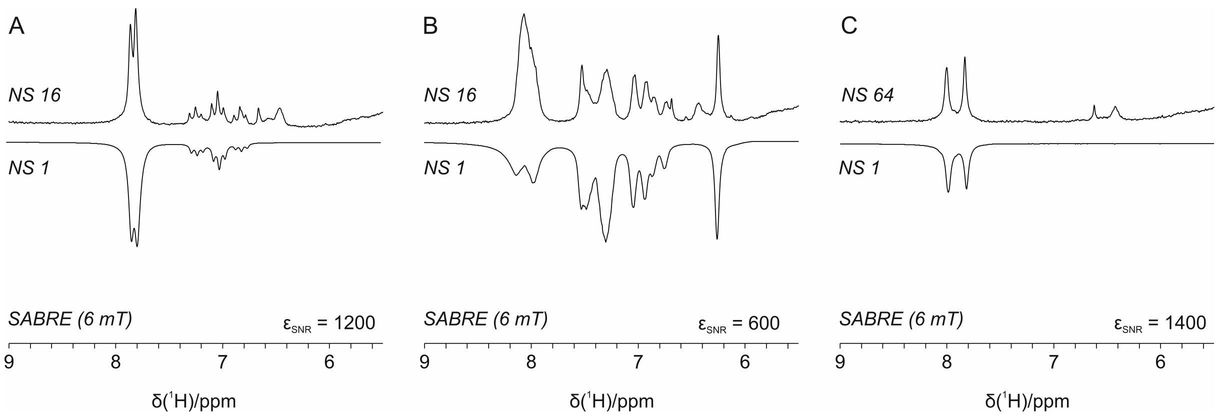


Figure S1: Comparison of reference thermally polarised (top) and SABRE-enhanced (bottom) $^{1}$H benchtop (1 T) NMR spectra of 100 mM of (A) **1**, (B) **2**, and (C) **3** with 5 mM SABRE pre-catalyst in methanol. The SNR enhancement factor ($\varepsilon_{SNR}$) was calculated as the signal-to-noise ratio of the hyperpolarised spectrum divided by that of the reference thermally-polarised spectrum. The signal-to-noise ratios were calculated as the maximum peak height divided by the standard deviation of the noise of a signal-free region of the spectrum. Where reference spectra were acquired using multiple scans, a SNR per single scan value was calculated by dividing the SNR by the square root of the number of scans.

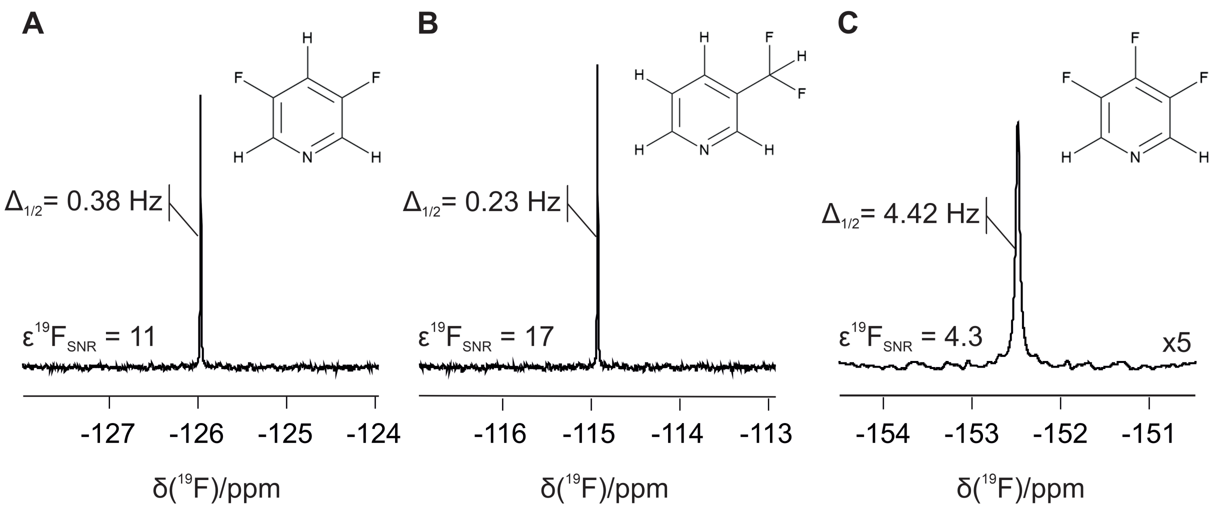


Figure S2: Reference SHARPER $^{19}$F benchtop (1 T) NMR spectra of 100 mM of (A) **1**, (B) **2**, and (C) **3** with 5 mM SABRE pre-catalyst in methanol. Full width at half maximum values for the SHARPER spectra include the effects of the matched filter. $\varepsilon_{SNR}$ values for **1**, **2**, and **3** are 11, 17 and 4.3 respectively. The SHARPER spectra were acquired using the pulse sequence in Figure 3A with $\varphi_1$= 2x,2(-x),2y,2(-y); $\varphi_2$ = 2(y,-y),2(x,-x); $\psi$ = $\varphi_1$.

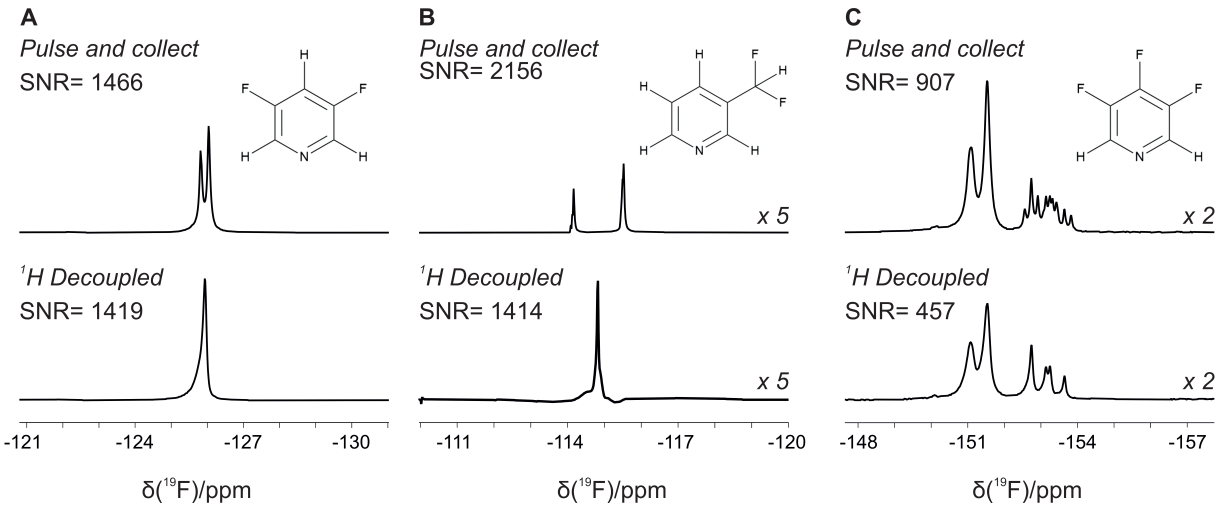


Figure S3: Comparison of SABRE-enhanced $^{19}$F benchtop (1 T) NMR spectra of 100 mM of (left) **1**, (centre) **2**, and (right) **3** with 5 mM SABRE catalyst in methanol. (top) Standard $^{19}$F SABRE acquisition; (bottom) $^{19}$F{$^{1}$H} SABRE acquisition.

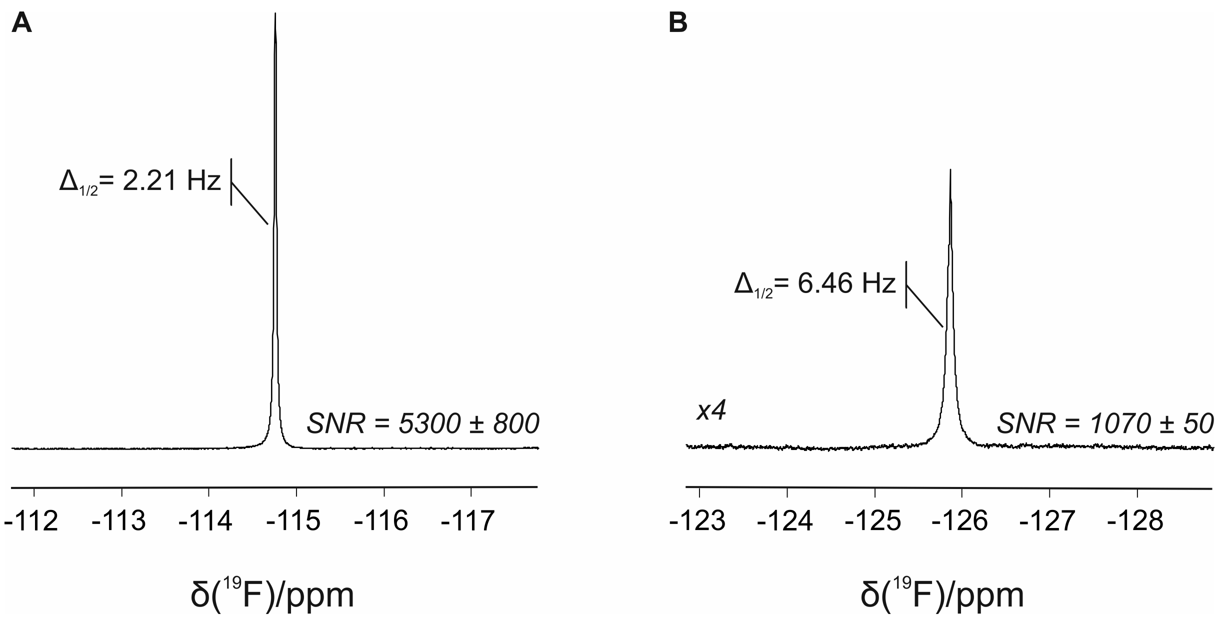


Figure S4: SABRE-*sel*SHARPER experiments on a mixture of A) **2** and B) **1** (50 mM of each) with 5 mM of catalyst in methanol. The *sel*SHARPER experiments were performed using selective pulses inside the loop. SABRE spectra were acquired with (A) PTF = 6.2 mT and (B) PTF $\sim$ 50 $\mu$T. Full width at half maximum values for the SHARPER spectra include the effects of the matched filter.

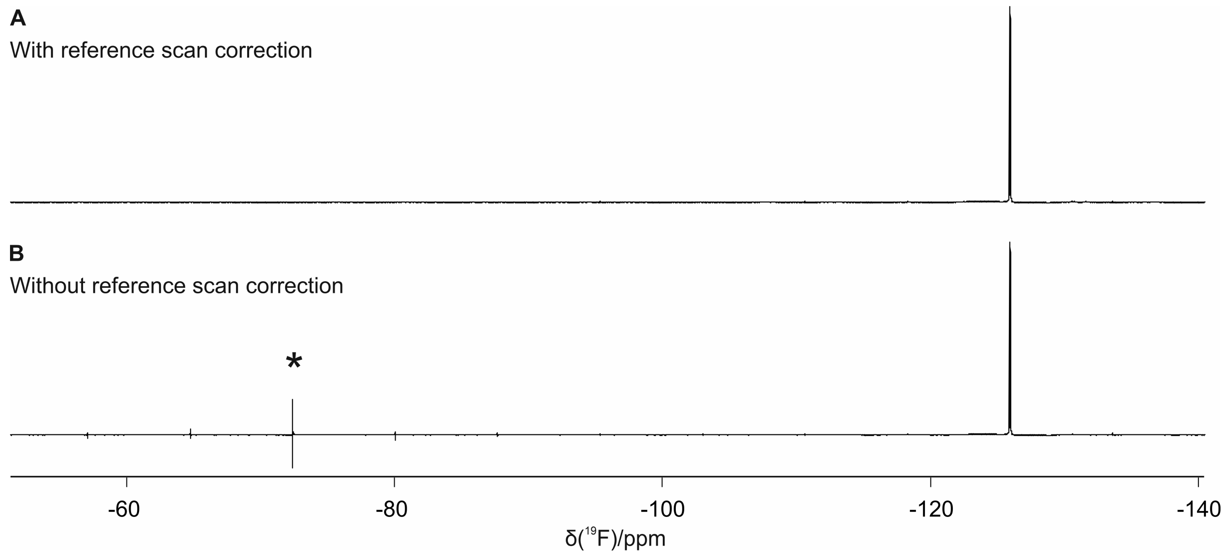


Figure S5: Comparison of $^{19}$F SABRE-SHARPER spectra acquired with (top) and without (bottom) a second reference scan to remove the effect of off-resonance artefacts that originate from background $^{19}$F signals from the probe at -72 ppm (shown with an asterisk). The reference scan is acquired with a change in phase of the refocusing pulses in the loop by 180°

.

# S5 Implementation of Gaussian pulse with RF non-linearity correction

The Magritek Spinsolve 43 Carbon spectrometer used in this work has a non-linear RF power output that leads to discrepancies between the RF pulse amplitude value that is set in the pulse program macro and the actual RF pulse amplitude that is outputted by the RF amplifier. The non-linear response of the RF amplifier was corrected to ensure an accurate formation of the selective pulses. Using a 90% $D_2O$ : 10% $H_20$ reference sample, $^1$H RF nutation curves with hard pulses were acquired for a range of user-defined RF amplitudes in dB, denoted $dB_{set}$. The RF pulse duration that corresponds to a $\pi/2$ nutation of the spins, $t^{exp}_{\pi/2}$, was calculated for each $dB_{set}$ value. These values were converted to an observed power level, denoted $dB_{ideal}$, using Eq. S1, where $t^{ref}_{\pi/2}$ is the duration of a $\pi/2$ pulse at 0 dB.

$$dB_{ideal} = 20\ \log_{10}\left(\frac{t^{ref}_{\pi/2}}{t^{exp}_{\pi/2}}\right) \qquad \text{(S1)}$$

Non-linear behaviour of the RF amplifier is observed when $dB_{set}$ is plotted against $dB_{ideal}$ as in FIGURE S6a. A $5^{th}$ order polynomial representation was found to provide a good fit to the data. The polynomial coefficients are tabulated in Table S6.

$$y = P_5\,x^5 + P_4\,x^4 + P_3\,x^3 + P_2\,x^2 + P_1\,x \qquad \text{(S2)}$$

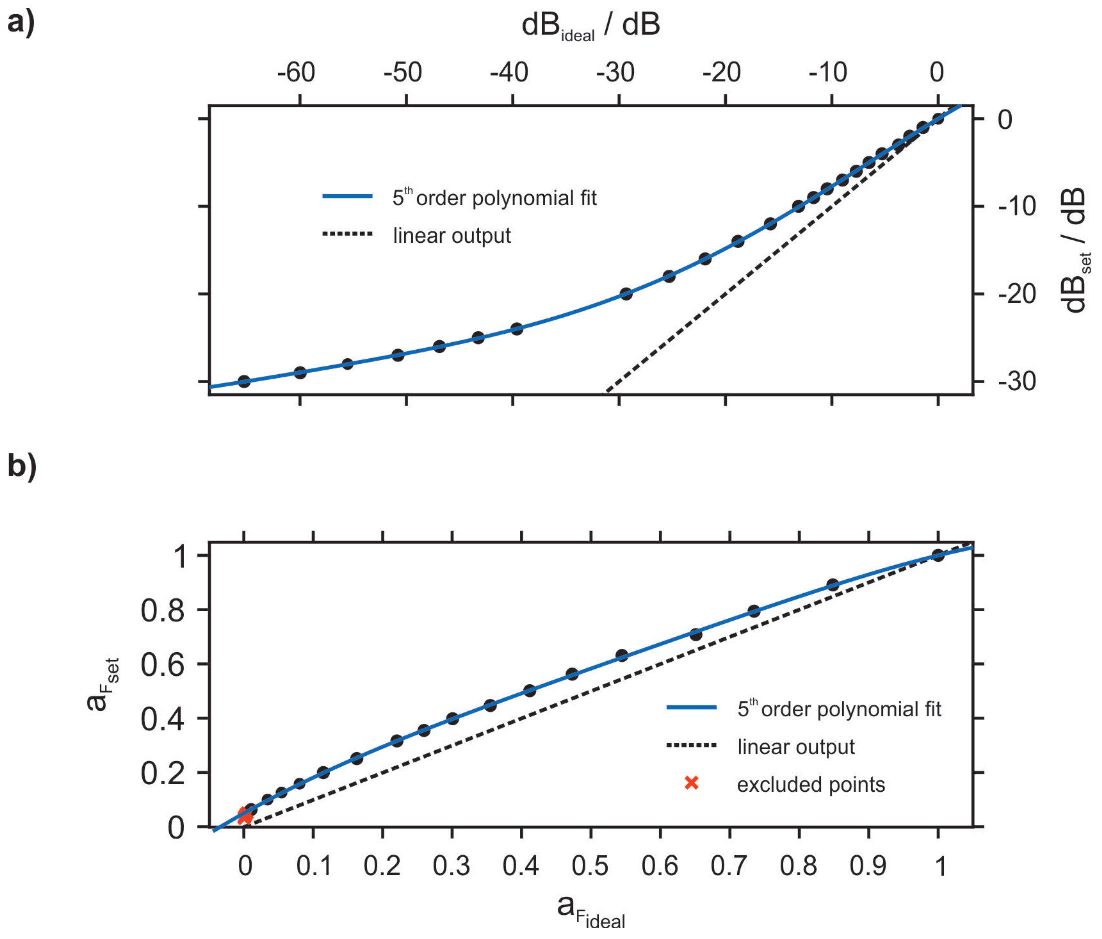


Figure S6: Calibration curve showing the non-linear power output of the RF amplifier in the a) dB scale and b) linear scale. Both sets of data points were fit to a 5th order polynomial that can be used to calculate the amplitude value to be set on the spectrometer, $dB_{set}$ or $a_{Fset}$, from the desired pulse amplitude, $dB_{ideal}$ or $a_{Fideal}$. The lowest 5 data points in b) were excluded from the fit because the RF amplitude cannot be accurately defined below a linear attenuation of 0.01. The equation and corresponding polynomial coefficients of the fit to the data in plot a) can be found in Eq. S2 and Table S6, and in plot b) can be found in Eq. S6 and Table S7, respectively.

Table S6: Table of coefficients of the $5^{th}$ order polynomial that is fit to the calibration curve of the RF synthesiser defined along a logarithmic (dB) attenuation scale.

| **Polynomial Coefficient** | **Value** |
|---|---|
| $P_1$ | 0.7365 |
| $P_2$ | $-8.224\times10^{-3}$ |
| $P_3$ | $-5.414\times10^{-4}$ |
| $P_4$ | $-7.771\times10^{-6}$ |
| $P_5$ | $-3.679\times10^{-8}$ |

The curve deviates significantly from a linear 1:1 relationship, as highlighted by the dashed black line. Although the fit is non-linear, it allows conversion from the $dB_{ideal}$ value to the $dB_{set}$ value. In other words, the polynomial provides the value that should be set on the pulse program macro in order for the RF amplifier to output the desired RF amplitude, thus correcting for the non-linear behaviour.

$$G = b^{(4\,x^2)} \qquad \text{between } -0.5 \leq x \leq 0.5 \qquad \text{(S3)}$$

The shape of the Gaussian pulse is outlined by tabulated values in the pulse program macro of the Spinsolve Expert software. These values are outputted one at a time by the RF amplifier over the duration of the pulse. To define the Gaussian shape, a table of values are generated using Eq. S3, which produces a Gaussian curve with a maximum amplitude of $G = 1$ at $x = 0$, and a cut-off amplitude at $x = \pm\,0.5$ determined by the parameter $b$, which is quoted as a percentage of the maximum amplitude and controls the width of the curve. It is important to note that the size of the table of $G$ values is dictated by the number of $x$ values used to describe the Gaussian pulse (see Eq. S3), where more $x$ values leads to a more accurate representation of the Gaussian shape. The duration of the RF pulse is also controlled by the number of $x$ values since the RF amplifier takes a minimum of $10\,\mu$s to step through each tabulated value. Therefore, a Gaussian defined by 100 $x$ values has a duration of $1000\,\mu$s ($100 \times 10\,\mu$s).

In order for the RF amplifier to output the Gaussian pulse, amplitude values must be represented as 14-bit numbers. A value of 0 corresponds to the highest possible attenuation of the RF pulse and a value of $2^{14}$-1 (16383) corresponds to a non-attenuated RF pulse (0 dB). Representation of the Gaussian amplitude values as 14-bit numbers is achieved within the pulse program macro in 3 steps. The first step requires converting the RF attenuation, which is defined by the user in dB ($dB_{set}$), into a linear attenuation factor, $a_{F_{set}}$.

$$a_{F_{set}} = 10^{\left(\frac{dB_{set}}{20}\right)} \tag{S4}$$

The values defining the Gaussian shape, $G$, are then multiplied by $a_{F_{set}}$ to attenuate each of them by the desired amount, producing a Gaussian curve with a maximum of $G_{att} = a_{F_{set}}$ at $x = 0$.

$$G_{att} = a_{F_{set}} G \tag{S5}$$

The second step requires the correction of these values ($G_{att}$) to overcome the non-linear output of the RF amplifier. The correction curve in Figure S6a was used to correct the values in dB, but cannot be used to correct the $G_{att}$ values which are defined along a linear scale. First we have to convert the dB attenuation values into linear attenuation factors to produce the correction curve in Figure S6b which plots set attenuation, $a_{F_{set}}$, against ideal attenuation, $a_{F_{ideal}}$. A $5^{th}$ order polynomial was fit to this data and is shown in Eq. S2, where the values of the polynomial coefficients are tabulated in Table S6. The corrected values, $G_{corr}$, were acquired by passing the attenuated values, $G_{att}$, through the polynomial. The fit is designed to fix the polynomial to coordinates (1,1), such that $G_{corr} = 1$ when $G_{att} = 1$. This is done to ensure no attenuation for an input value of 0 dB.

$$G_{corr} = p_5(G_{att} - 1)^5 + p_4(G_{att} - 1)^4 + p_3(G_{att} - 1)^3 + p_2(G_{att} - 1)^2 + p_1(G_{att} - 1) + 1 \tag{S6}$$

Table S7: Table of coefficients of the $5^{th}$ order polynomial that is fit to the calibration curve of the RF synthesiser defined along a linear attenuation scale.

| **Polynomial Coefficient** | **Value** |
|---|---|
| $p_1$ | 0.6281 |
| $p_2$ | -0.8736 |
| $p_3$ | -1.329 |
| $p_4$ | -0.8460 |
| $p_5$ | -0.0677 |

The Gaussian curve and its corrected version is illustrated in Figure S7 for $dB_{set}$ values of 0 and -12 dB. All Gaussian curves were generated with 1000 points in the $-0.5 \leq x \leq 0.5$ range, and with a cut-off amplitude of $b = 0.01$. The linear polynomial correction is seen to have a larger effect on the shape of the Gaussian at lower amplitudes as this is the region of the correction curve where the RF amplifier significantly deviated from linearity (see Figure S6).

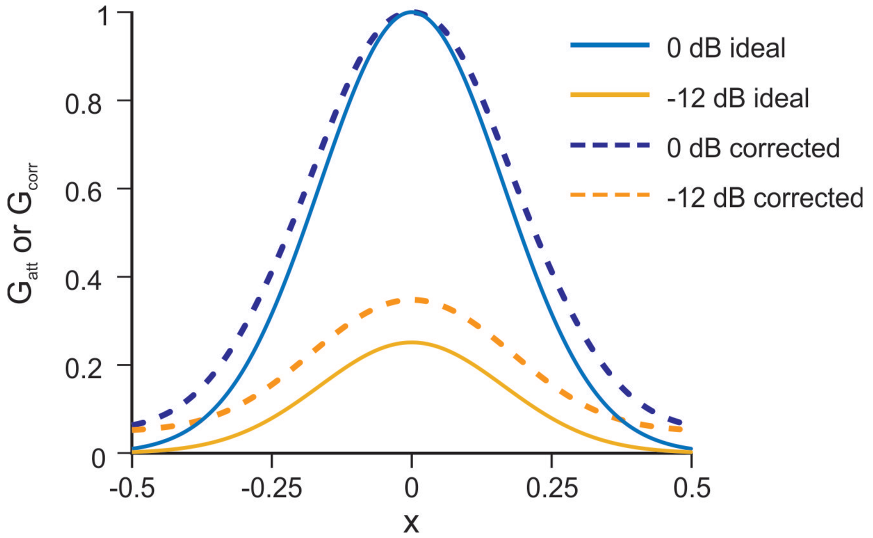


Figure S7: A plot of Gaussian curves with two different attenuations ($dB_{set}$) of 0 and -12 dB described by Eq. S3 using 1000 amplitude values and a cut-off amplitude of b = 0.01. The curves represented by solid and dashed lines depict the ideal and corrected versions of the Gaussian, respectively, where the corrected versions involve passing the $G_a$ values through the $5^{th}$ order polynomial fit in Eq. S2 from the linear correction curve in Figure S6b.

The third and final step takes the corrected values, $G_{corr}$, and multiplies them by 16383 ($2^{14}$-1) to represent them as 14-bit numbers that the spectrometer can interpret and use to produce the desired Gaussian RF pulse. All $^{19}$F Gaussian pulses were implemented using the same RF correction procedure. Gaussian inversion pulse calibration was achieved using a SPFG-SE (single pulsed-field gradient spin-echo) pulse sequence and determining the $a_F^{set}$ for which optimal signal was observed for a given pulse duration.
